# Supplementary material for: The effectiveness of botulinum toxin for temporomandibular disorders: A systematic review and meta-analysis
Source: PLoS One. 2024 Mar 14;19(3):e0300157. doi: 10.1371/journal.pone.0300157 (PMC10939295; doi:10.1371/journal.pone.0300157)
Supplement: Attachment — Submitted filename: Response Letter.docx [file pone.0300157.s003.docx]

| Dear Editor and Reviewers,  We appreciate your precious time in reviewing our paper and providing valuable comments. Your insightful words led to possible improvements in the current version. We have carefully considered the statements and tried our best to address every one of them. Besides, the entire text has been thoroughly revised to rectify English writing mistakes. We hope the manuscript, after careful revisions, meets your high standards. The authors welcome further constructive comments, if any. We provide point-by-point responses as follows. | |
| --- | --- |
| **REVIEWER 1** | **REPLIES** |
| Introduction: The introduction is lack of information regarding the possible mini-invasive treatment of TMD (e.g ozone-therapy, hyaluronic acid etc.). Please, read doi: 10.1111/joor.13571. | - Using the valuable study you provided, we have added information on additional management techniques like ozone therapy, platelet-rich plasma (PRP) therapy, and hyaluronic acid (HA) therapy. |
| Material and Methods: The authors have included in PICO model partecipants suffered TMD. How was the diagnosis performed? this point is crucial to better clarify the TMD . I suggest to read PMID: 20213030. | - Thank you for your comment. Additional data on the issue has been provided in the "Research Questions and Study Objective." |
| Moreover, I suggest to add in table a brief protocol strategy for BTX for each study with the muscle involved in the protocol therapy | - The requested data have been added in Appendix Table 1 under the column title "intervention group details." Thank you for this valuable suggestion. |
| Discussion: The discussion is a simple list of the results of the research. I suggest to rewrite it at light of the potential use of BTX in muscular and neuromuscular disease. | - The discussion section has been further improved according to your comment, looking at the possible impact of review findings on BTX use for TMD disorders in the future. |
| **REVIEWER 2** | **REPLIES** |
| **Abstract**  In my opinion the abstract should be revised in order to better describe the object of this Systematic Review, also describing controls and outcomes | - Done. The objective has been better worded to report the primary and secondary outcomes under study.  A report on control treatments has been provided in the table. The studies did not use the same control treatment; thus, we cannot word the objective regarding one control treatment. The meta-analysis was, however, done only for placebo injections as a control. We have made this clear in the abstract and results. |
| **Introduction**  - The reference should be placed in square brackets.  - In this section I suggest also to describe the various treatment possibility, including physical therapy and medications, other than splint.  - It is also necessary to underpin the muscle treated with botulinum toxin across the studies  - Lastly the objective should be revised in order to better include population, study selection, outcomes and controls, and should be written in scientific language ("The goal of this study is to determine if botulinum toxin can treat TMJ problems" seems too much colloquial for a systematic review with metanalysis)  - Botulinum toxin's possible action pathways in treating TMD should be describe also in this section (other than in discussion)  - Other medical condition related to TMD should be evaluated in this section  You might consider reading these recent works:  • Shimada A, Ishigaki S, Matsuka Y, Komiyama O, Torisu T, Oono Y, Sato H, Naganawa T, Mine A, Yamazaki Y, Okura K, Sakuma Y, Sasaki K.  Effects of exercise therapy on painful temporomandibular disorders. J Oral Rehabil. 2019 May;46(5):475-481. doi: 10.1111/joor.12770. Epub 2019 Feb 19. PMID: 30664815.  • Schiffman E, Ohrbach R, Truelove E, Look J, Anderson G, Goulet JP, List T, Svensson P, Gonzalez Y, Lobbezoo F, Michelotti A, Brooks SL, Ceusters W, Drangsholt M, Ettlin D, Gaul C, Goldberg LJ, Haythornthwaite JA, Hollender L, Jensen R, John MT, De Laat A, de Leeuw R, Maixner W, van der Meulen M, Murray GM, Nixdorf DR, Palla S, Petersson A, Pionchon P, Smith B, Visscher CM, Zakrzewska J, Dworkin SF; International RDC/TMD Consortium Network, International association for Dental Research; Orofacial Pain Special Interest Group, International Association for the Study of Pain. Diagnostic Criteria for Temporomandibular Disorders (DC/TMD) for Clinical and Research Applications: recommendations of the International RDC/TMD Consortium Network* and Orofacial Pain Special Interest Group†. J Oral Facial Pain Headache. 2014 Winter;28(1):6-27. doi: 10.11607/jop.1151. PMID: 24482784; PMCID: PMC4478082.  • Ferrillo M, Lippi L, Giudice A, Calafiore D, Paolucci T, Renò F, Migliario M, Fortunato L, Invernizzi M, de Sire A. Temporomandibular Disorders and Vitamin D Deficiency: What Is the Linkage between These Conditions? A Systematic Review. J Clin Med. 2022 Oct 22;11(21):6231. doi: 10.3390/jcm11216231. PMID: 36362456; PMCID: PMC9655046.  • Gil-Martínez A, Paris-Alemany A, López-de-Uralde-Villanueva I, La Touche R. Management of pain in patients with temporomandibular disorder (TMD): challenges and solutions. J Pain Res. 2018 Mar 16;11:571-587. doi: 10.2147/JPR.S127950. PMID: 29588615; PMCID: PMC5859913. | -All in-text citations have been reformatted.  - We have added information on additional management techniques like ozone therapy, platelet-rich plasma (PRP) therapy, and hyaluronic acid (HA) therapy. Thank you for your suggestion.  - The requested data have been added in Appendix Table 1 under the column title "intervention group details." Thank you for this valuable suggestion.  -Done. The objective has been better worded to report the primary and secondary outcomes under study.  -Reports on control treatments have been provided in the table. The studies did not use the same control treatment; thus, we cannot word the objective regarding one control treatment.  -the meta-analysis was, however, done only for placebo injections as a control. I have made this clear in the abstract and results.  -The requested information has been added to the corresponding text within the introduction.  -Thank you for your recommendation. However, we did not go into detail about TMD-related conditions because we wanted the study to be specific to TMD only. We have, however, reported on the conditions that present due to the underlying issue of TMD.  Thanks for your valuable input. All these references provided very useful information and have been added in the references 52.53.54.55.  Done  Done  Done  Done |
|  |  |
| **Materials and Methods**  -In this section i suggest to separate the primary outcome and the preferred scale evaluation (VAS), from the secondary outcomes.  - Was the string utilized also on "Dimension Publication"?  - "Clinical trials without a population could only be eligible if the results for the adult population were reported separately" this section should be revised to better explain the study selection  - "The trial's primary objective had to be examining the effectiveness of BTX in managing TMD" the objective of the systematic review seems to be the effectiveness of BTX in treating pain in patients with TMD, as the effectiveness is not a quantifiable outcome and authors utilized VAS score | -It has been revised accordingly.  -The string used in Dimension Publication has been added to the search string table.  - It has been revised accordingly.  -This had been written better under the section "Research Questions and Study Objective." |
| **Results**  - In the subheading describing the population, the ratio male /female and other discrete variables should be explicated as percentages, while continuous variables should be written as mean and standard deviation  - in this section I suggest to better compare the various control treatments between the studies  - The metanalysis for VAS only include 2 to 3 studies at time, as it might be inconclusive for this type of study. I have also doubts regarding the adverse events analysis, where RR was 1,34 times higher for placebo group at one month compared with botulinum toxin  - author stated that "Nevertheless, the statistical analysis revealed that there were no significant mean differences seen between the groups receiving BTX-A and placebo across all timeframes. This indicates that the administration of BTX-A did not yield superior outcomes compared to the absence of treatment in terms of pain reduction among patients with temporomandibular disorder (TMD) at 1, 3, and 6 months" this section is in contrast with all the paper until that, and should be better explained. | -In the table, under the column heading 'participants', the male/female is reported as a percentage of the entire population, age is reported as mean, and standard deviation is provided. Some studies reported only the mean, and others only reported the age range.  -In the results and discussion sections, we have reported how other treatments, e.g., facial manipulation, control treatment, and laser therapy, faired compared to placebo and each other.   - I appreciate your consideration. In the discussion section, we addressed the fact that this result is inconclusive.   -Any contradictions that might have arisen from this sentence have been resolved by improved reporting on the abstract, results, and discussion sections. |
| **Discussion**  - Study limitation should be implemented according to this revision  - In Author's contribution name and surname should be pointed  - Lastly, references are only partly formatted in accordance with this Journal instruction for Authors. | - It has been revised accordingly.  - It has been revised accordingly.  - They have been revised accordingly. |
